# Anti-Obesity Effects of Aqueous Extracts of Sunbanghwalmyung-Eum in High-Fat- and High-Cholesterol-Diet-Induced Obese C57BL/6J Mice

**DOI:** 10.3390/nu14142929

**Published:** 2022-07-17

**Authors:** Hye-Lin Kim, You Mee Ahn, So Min Lee, Chang-Seob Seo, Seong-Hwan Park, Ok-Sun Bang, Jeeyoun Jung

**Affiliations:** 1 KM Science Research Division, Korea Institute of Oriental Medicine, Daejeon 34054, Korea; hlkim@kiom.re.kr (H.-L.K.); 2hyunm@kiom.re.kr (Y.M.A.); csseo0914@kiom.re.kr (C.-S.S.); ppjksh91@kiom.re.kr (S.-H.P.); osbang@kiom.re.kr (O.-S.B.); 2 KM Convergence Research Division, Korea Institute of Oriental Medicine, Daejeon 34054, Korea; dasonya@kiom.re.kr

**Keywords:** Sunbanghwalmyung-eum, high-fat high-cholesterol diet, obesity, C57BL/6J mice

## Abstract

Sunbanghwalmyung-eum (SBH) is a traditional herbal medicine that exhibits various pharmacological properties, such as antioxidant, anti-inflammatory, and anticancer activities. In this study, we investigated the systemic anti-obesity effects of an aqueous extract of SBH in the liver, adipose, and muscle tissue from high-fat and high-cholesterol diet (HFHCD)-induced obese C57BL/6J mice. After 6 weeks of an HFHCD, the mice were continuously fed HFHC with oral administration of SBH (100 mg/kg/day), Sim (simvastatin, 5 mg/kg/day, positive control), or water (HFHC only) for another 6 weeks. Our results showed that SBH attenuated the HFHCD-induced body weight gain and fat accumulation in the liver, and improved plasma lipid levels, such as those of triglycerides (TGs), blood total cholesterol (TC), and low-density lipoprotein (LDL-c). SBH and Sim inhibited the inflammation accompanied by obesity via decreasing inflammatory cytokine interleukin (IL)-1β, tumor necrosis factor α (TNFα), and monocyte chemoattractant protein 1 (MCP1). Moreover, SBH downregulated the expression of protein levels of adipogenic-related factors, including peroxisome proliferator-activated receptor γ (PPARγ) and CCAAT/enhancer-binding protein α (C/EBPα), in the liver, adipose, and muscle tissue. The SBH and Sim treatment also significantly upregulated the phosphorylation of AMP-activated protein kinase α (AMPKα) in the liver and hormone-sensitive lipase (HSL) in the adipose tissue. Overall, the effects of SBH on HFHCD-induced obesity were similar to or more potent than those of simvastatin. These results indicated that SBH has great potential as a therapeutic herbal medicine for obesity.

## 1. Introduction

Obesity contributes to many health-related problems and is an independent risk factor for metabolic syndrome linked to other disorders, including cardiovascular disease, type 2 diabetes mellitus (T2D), hypertension, dyslipidemia, and cancer [1,2]. Along with the globalization of the Western diet, a high-fat and high-cholesterol diet (HFHCD) is considered one of the most important causes of the high incidence of obesity and hyperlipidemia worldwide [3]. Therefore, the establishment of animal models with an HFHCD may be useful for evaluating anti-obesity potential for the treatment of obesity and related complications.

Adenosine 5-monophosphate-activated protein kinase (AMPK) maintains energy homeostasis and is involved in the energy metabolism of organs, including the liver, adipose tissue, and muscle. Additionally, it regulates the expression and phosphorylation of enzymes and genes involved in glucose and lipid metabolism [4]. Phosphorylated AMPK inhibits metabolic enzymes involved in the regulation of fatty acid synthesis and fat production. AMPK is also known to play an anti-inflammatory role in adipocytes by regulating pro-inflammatory factors, which can inhibit obesity-induced insulin resistance [5].

Adipogenesis is the developmental process of adipocyte precursor cells in mature adipocytes. The regulation and understanding of the molecular mechanism of adipogenesis might present a possible therapeutic approach for obesity. Adipogenesis is regulated by transcriptional cascade and signaling pathways, such as the peroxisome proliferator-activated receptor γ (PPARγ) and cytidine-cytidine-adenosine-adenosine-thymidine (CCAAT) enhancer-binding protein alpha (C/EBPα) [6]. These molecules are the major transcriptional regulators of lipid homeostasis, which regulate fat accumulation and lipolysis [7]. In addition, excessive body fat promotes the secretion of interleukin (IL) and tumor necrosis factor alpha (TNFα), thereby increasing the inflammatory response, inhibiting insulin signal transduction, and inducing insulin resistance [8]. Thus, regulation of these obesity-related factors and their signaling molecules may provide an alternative to current approaches for treating obesity. Due to the global increase in the number of obese patients, various pharmacological drugs have been developed to treat obesity [9]. Simvastatin, which is one of the statin drugs, is a lipid-lowering agent that inhibits HMG-CoA reductase and has been used to treat dyslipidemia and cardiovascular diseases [10,11]. Therefore, it was chosen as a positive control in the HFHCD-induced model. However, although statin drugs cause side effects, such as joint pain, memory loss, and myopathy, natural products have relatively few side effects and low toxicity, making them an attractive alternative treatment for obesity [12].

One of them, namely, Sunbanghwalmyung-eum (SBH, Xian-Fang-Huo-Ming-Yin, XFHM), is a modified formula mentioned in Jiaozhu Furen Liangfang, which was compiled by Xue Ji during the Chinese Ming Dynasty. It has been used for hundreds of years to treat many diseases, such as sores and carbuncles [13]. Some studies showed that SBH can be used to treat osteoarthritis, as well as showing that SBH has antioxidant, anti-inflammatory, and anti-cancer properties [14,15]. Although various efficacies of SBH were shown as described above, efficacy studies on chronic metabolic diseases are insufficient. Therefore, this study was conducted to examine the anti-obesity effects of SBH in HFHCD-induced obese mice.

## 2. Materials and Methods

### 2.1. Chemicals and Reagents

Gallic acid (99.5%), aloe-emodin (95.0%), and rhein (technical grade) were purchased from Merck KGaA (Darmstadt, Germany). Hesperidin (98.6%) and emodin (98.2%) were purchased from Biopurify Phytochemicals (Chengdu, China). Chlorogenic acid (99.6%), paeoniflorin (98.8%), and oxypeucedanin hydrate (98.0%) were purchased from Acros Organics (Pittsburgh, PA, USA), Wako Chemicals (Osaka, Japan), and ChemFaces Biochemical Co., Ltd. (Wuhan, China), respectively. HPLC-grade solvents (methanol, acetonitrile, and water) for the dissolution and chromatographic separation of marker compounds were obtained from J. T. Baker (Phillipsburg, NJ, USA). Trifluoroacetic acid (TFA, for HPLC, ≥99.0%) was obtained from Merck KGaA (Darmstadt, Germany).

### 2.2. Preparation of SBH Extract and HPLC Analysis of SBH

With a slightly modified XFHM prescription, SBH was prepared as an aqueous extract from a mixture of the following 13 dried medicinal herbs: *Glycyrrhiza uralensis* Fischer, *Lonicera japonica* Thunberg, *Angelica gigas* Nakai, *Rheum palmatum* Linne, *Commiphora myrrha* Engler, *Saposhnikovia divaricata* Schischkin, *Angelica dahurica* Bentham et Hooker f., *Paeonia lactiflora* Pallas, *Gleditsia japonica* Miquel var. koraiensis Nakai, *Citrus unshiu* Markovich, *Fritillaria ussuriensis*, *Trichosanthes kirilowii* Maximowicz, and sulfur. All herbal materials were purchased from Omniherb (Daegu, Korea) in 2016. The herbal mixture was refluxed twice in distilled water (10 L/kg) for 3 h, and the extract was filtered, concentrated using a rotary evaporator, and dried using a freeze drier. The voucher specimen (no. BS5-1) and each herbal component were stored at the Korea Institute of Oriental Medicine (KIOM, Daejeon, Korea). The final aqueous extract of SBH was obtained as a powder and stored at −20 °C until use. An analysis of the quality of the SBH sample was conducted using an LC-20A Prominence HPLC system (Shimadzu Co., Kyoto, Japan) consisting of two pumps, an online degasser, a column oven, an autosampler, a PDA detector, and a data processing unit that used LabSolution software (Version 5.54 SP3). The analytical column that was used for the separation of major components in the SBH was a SunFire C_18_ column (4.6 × 250 mm, 5 μm, Milford, MA, USA), and the column temperature was maintained at 40 °C. All analytes, including the eight standards, were separated using a sequential gradient mobile phase system from a 95:5 mixture of distilled water (mobile phase A) and acetonitrile (mobile phase B), both containing 0.1% (*v*/*v*) TFA, to a 40:60 mixture in 40 min, and to a 100:0 mixture in 50 min, at a flow rate of 1.0 mL/min. For the quantitative analysis of SBH using HPLC-PDA, 200 mg of the lyophilized SBH was dissolved in 20 mL of 70% methanol and extracted for 60 min using an ultrasonicator (Branson 8510E-DTH, Denbury, CT, USA). After the extraction, the extracted solution was filtered using a 0.2 μm membrane filter (PALL Life Sciences, Ann Arbor, MI, USA) before the HPLC injection.

### 2.3. Animal Experiment

All animal treatment and handling procedures were approved by the Institutional Animal Care and Use Committee of the Korea Institute of Oriental Medicine (IACUC no. 16-044). Six-week-old male C57BL/6J mice (Samtako Inc., Osan, Korea) were used in the present experiments. The mice were housed in a specific pathogen-free facility and maintained under specific pathogen-free (SPF) laboratory conditions in a 12/12 h light-dark cycle, a constant temperature of 22 ± 3 °C, and relative humidity of 45–60%. After a two-week adjustment period, the mice were divided into two groups: a normal chow diet (Nor group, *n* = 8; D12450B) or an HFHC diet (HFHC group, 5TJT). After 6 weeks, the HFHC diet (HFHCD) mice were randomly divided into three groups and then fed with HFHC (HFHC group, *n* = 7), simvastatin (Sim group, 5 mg/kg/day, *n* = 7) as a positive control, or SBH (SBH group, 100 mg/kg/day, *n* = 7) for an additional 6 weeks. The body weights of the mice were measured weekly. At the end of the experiment, the animals fasted for 4 h and blood was collected from the abdominal aorta of mice anesthetized with isoflurane. Blood was collected in pre-chilled tubes containing 75 USP units of heparin (Becton, Dickinson and Company, Franklin Lakes, NJ, USA). Liver, kidney, and adipose tissues (epididymal adipose tissue) samples were isolated, snap frozen in liquid nitrogen, and stored at −80 °C until use.

### 2.4. Plasma Biochemical Parameter Analysis

Plasma was separated immediately after the blood sampling via centrifugation at 4000× *g* for 30 min. TC, TG, HDLc, GOT, and GPT were measured using dry methods and solid-phase reagents (SPOTCHEM^TM^ EZ (ARKRAY, Kyoto, Japan)) according to the manufacturer’s instructions. The estimated LDLc was obtained using the Friedewald equation method, which follows Equation [16]:

$$\mathrm{LDLc}\;\left(\mathrm{mg}/\mathrm{dL}\right)=\;\mathrm{TC}-\mathrm{HDLc}-\frac{TG}{5}$$

GIP, GLP-1, and insulin levels were measured using the Bio-Plex Pro Mouse Diabetes 8-plex Assay (Bio-Rad Laboratories, Hercules, CA, USA). Samples were read using the Bio-Plex 200 System with Bio-Plex Manager software (Bio-Rad Laboratories, Hercules, CA, USA).

### 2.5. OGTT

The OGTT was performed after overnight fasting for 10 weeks of the experimental period. Glucose was orally administered to mice at a concentration of 2 g/kg body weight, and blood was collected from the tail vein at 0, 20, 40, 60, and 120 min after a glucose injection. Blood glucose levels were measured using an Accu-Chek meter (Roche, Basel, Switzerland).

### 2.6. H&E Staining

Liver tissue from mice was fixed by using 4% formalin, dehydration, and embedding in paraffin (Leica, Wetzlar, Germany), where the blocks were cut into 4 µm sections and stained with hematoxylin and eosin (H&E). Images were analyzed using the ImageJ (NIH, Bethesda, MD, USA) program [17].

### 2.7. Western Blot Analysis

Western blot analysis was performed as previously described [18]. Homogenized tissues were lysed in an ice-cold RIPA buffer, and protein concentrations were determined using a protein assay reagent (Bio-Rad Laboratories, Hercules, CA, USA). Equal amounts of protein (30–50 µg) were resolved using 4–12% sodium dodecyl sulfate-polyacrylamide gel electrophoresis (SDS-PAGE) and transferred to a polyvinylidene difluoride membrane. The membranes were incubated overnight at 4 °C with primary antibodies (Table 1) and then incubated with secondary antibodies conjugated to horseradish peroxidase (Enzo Life Sciences, Farmingdale, NY, USA) for 2 h. The bands were visualized using enhanced chemiluminescence (Bio-Rad Laboratories Inc., Hercules, PA, USA).

### 2.8. Statistical Analysis

All data are expressed as the mean ± standard error of the mean (SEM) of three or more experiments. The significance of results was performed using one-way or two-way analysis of variance (ANOVA) followed by Tukey’s multiple comparison test, using GraphPad Prism version 8 (GraphPad software, La Jolla, CA, USA).

## 3. Results

### 3.1. Quantification of the Eight Marker Compounds in SBH

**Figure 1:**
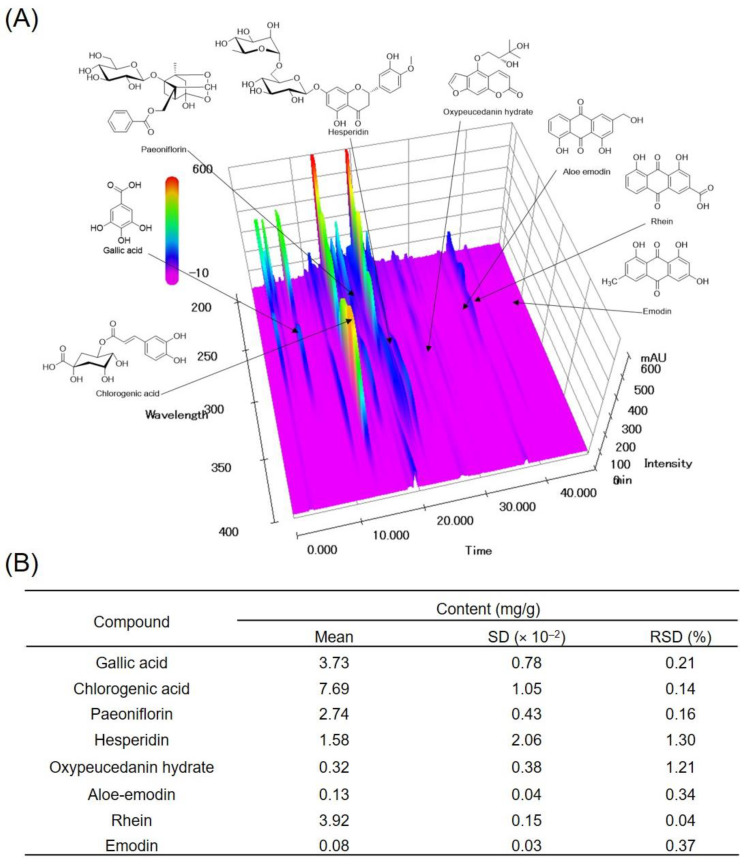
Three-dimensional HPLC-PDA chromatogram (**A**) and the contents of eight marker components in SBH. Repeatability was confirmed by the RSD (RSD (%) = standard deviation (SD)/mean × 100%) (**B**). SD, standard deviation; RSD, relative standard deviation.

SBH was prepared via water extraction with a 23.03% yield, and its quality was verified using a high-performance liquid chromatography-photodiode array (HPLC-PDA) system. The three-dimensional HPLC profile of the SBH is shown in Figure 1. The retention times of eight major compounds, namely, gallic acid, chlorogenic acid, paeoniflorin, hesperidin, oxypeucedanin hydrate, aloe-emodin, rhein, and emodin, in the SBH chromatogram were 6.50, 13.62, 17.22, 21.55, 27.21, 36.32, 37.75, and 45.01 min, respectively (Figure 1A). The coefficients of determination (*r*^2^) of the calibration curves for the eight compounds and standard chemicals were 0.9999–1.0000, indicating good linearity in the optimized concentration ranges. The limit of detection (LOD) and limit of quantification (LOQ) of the eight compounds were 0.05–0.29 and 0.14–0.88 μg/mL, respectively. The contents of eight marker components in the SBH were analyzed using the optimized HPLC-PDA analytical method, and the SBH content was detected as 0.08–7.69 mg/lyophilized g (Figure 1B).

### 3.2. SBH Reduced HFHCD-Induced Body Weight Gain

**Figure 2:**
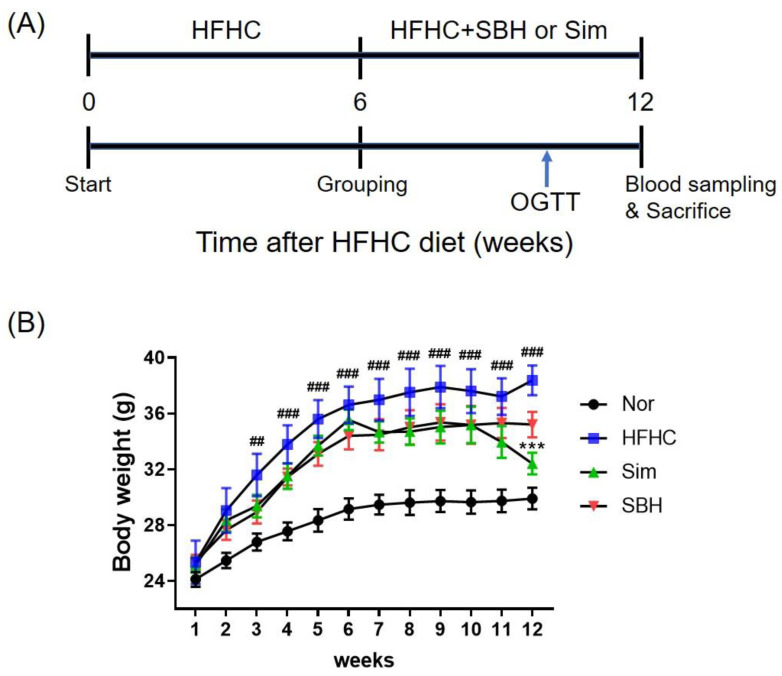
Effect of SBH on the changes in body weights in HFHCD-induced obese mice. Male mice were fed normal chow (Nor, *n* = 8) or HFHCD for 6 weeks, and then were administered HFHCD with Sim as a positive control (Sim, 5 mg/kg/d, *n* = 7), SBH (100 mg/kg/d, *n* = 7), or a vehicle (HFHC only, *n* = 7), as depicted in the experimental schedule (**A**). Body weights of the experimental mice were measured every week during the entire experimental period (**B**). Significant differences between the groups were assessed using two-way ANOVA with Tukey’s multiple comparison test. ^##^ *p* < 0.01, ^###^ *p* < 0.001 compared with Nor; *** *p* < 0.001 compared with HFHC. Nor, normal control group; HFHC, high-fat and high-cholesterol diet group; Sim, HFHC diet plus Sim group; SBH, HFHC diet plus SBH group.

We first developed an animal model of obesity to investigate the effects of SBH on obesity. As depicted in Figure 2A, C57BL/6J mice were fed a normal chow diet (normal) or a high-fat and high-cholesterol diet. After 6 weeks, the HFHCD mice were divided into three groups and each group received either an HFHCD (HFHC group), HFHC plus SBH (SBH group, 100 mg/kg), or HFHC plus Sim (Sim, positive control, 5 mg/kg/day) for a further 6 weeks. The HFHCD mice gained more weight up to 13.03 ± 1.06 g (32.6%) than the gain weight of 9.82 ± 0.91 g of the normal chow diet group after 12 weeks. Herein, the Sim and SBH treatments decreased the body weight gains compared with those of the HFHC mice. In particular, the mean weight of the Sim mice was significantly lower than that of the HFHC mice at the end of the experimental period (Figure 2B, *p* < 0.001).

### 3.3. SBH Reduced HFHCD-Induced Liver Disorder Factor and Lipid Parameters

To investigate obesity-induced liver-related risk, liver, kidney, and adipose tissues were isolated, and their weights were measured after 12 weeks of the experiment. The weights of the liver and adipose tissue were significantly increased in the HFHCD mice, which was significantly decreased by the administration of Sim or SBH. However, there was no significant difference in kidney weight between the four groups (Table 2).

**Figure 3:**
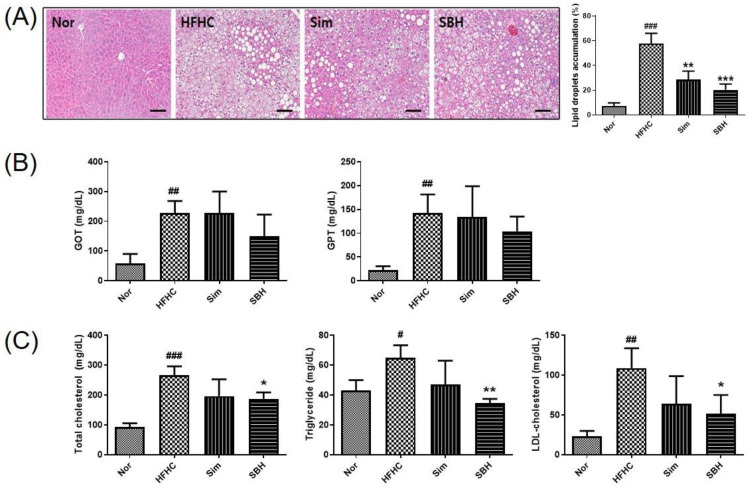
Effect of SBH on the histological change of liver tissue and the plasma levels of lipid parameters. Histological images of liver tissues from representative mice in each group (**A**). Tissues were fixed, embedded in paraffin, and stained with hematoxylin and eosin (H&E). Images are shown at the original magnification of ×200, and the scale bar is 1 mm. Quantification of lipid droplet area in the experimental animals. Data are expressed as a percentage of the area of lipid droplets in the field. The levels of plasma GOT and GPT (**B**) and the total cholesterol, triglyceride, and LDL-cholesterol were measured (**C**). The number of experiments: Nor (*n* = 4), HFHC (*n* = 4), Sim (*n* = 4), SBH (*n* = 4). Data are expressed as the mean ± SEM. ^#^ *p* < 0.05, ^##^ *p* < 0.01, ^###^ *p* < 0.001 compared with Nor; * *p* < 0.05, ** *p* < 0.01, *** *p* < 0.001 compared with HFHC. Nor, normal control group; HFHC, high-fat high-cholesterol diet group; SBH, HFHC diet plus SBH group; Sim, HFHC diet plus Sim group.

Hematoxylin and eosin (H&E) staining was performed to evaluate histological changes, such as lipid accumulation in tissues (Figure 3A). Steatosis and hepatocyte bal-looning were found in the liver tissue of HFHCD mice, which was significantly reversed in liver tissue from the SBH- or Sim-administered mice. The results indicated that the area of lipid accumulation in the liver was significantly increased in the HFHCD mice, which was significantly decreased by Sim or SBH administration. Furthermore, HFHCD induced a significant increase in plasma levels of glutamate oxaloacetate transaminase (GOT) and glutamate pyruvate transaminase (GPT) (Figure 3B). These factors are hepatoxicity related and their levels in plasma are a valuable aid in the diagnosis of liver disease [19]. Sim administration did not affect the levels of GOT and GPT in the HFHCD-induced mice, while SBH administration showed a tendency to decrease the GOT and GPT levels, but it was not significant. As a result of the analysis of plasma lipid content, HFHCD-induced mice had significantly increased plasma total cholesterol (TC), triglyceride (TG), and low-density lipoprotein (LDL)-cholesterol (LDLc) content compared with the Nor mice (Figure 3C). Herein, SBH administration significantly reduced the plasma lipid contents. Meanwhile, there was no significant difference in Sim administration. Levels of high-density lipoprotein (HDL)-cholesterol (HDLc) were also higher in the HFHCD-induced mice than in the Nor mice, but there was no difference Sim and SBH. These results indicated that SBH administration ameliorated HFHCD-induced hepatic toxicity and hyperlipidemia.

### 3.4. SBH Suppressed HFHCD-Induced Glucose Tolerance and Plasma Biomarkers of Diabetes

**Figure 4:**
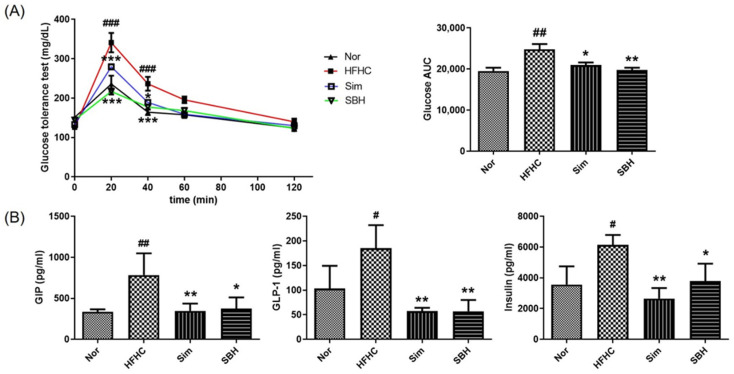
Effects of SBH on glucose tolerance and plasma biomarker concentrations related to insulin secretion. Oral glucose tolerance test (OGTT) and fasting blood glucose levels in C57BL/6J mice were measured. The area under the curve (AUC) was determined using the trapezoidal rule (**A**). The levels of GIP, GLP-1, and insulin were measured using the Bio-Plex Pro Mouse Diabetes 8-plex Assay (**B**). The number of experiments: Nor (*n* = 4), HFHC (*n* = 4), Sim (*n* = 4), SBH (*n* = 4). Significant differences between the groups were assessed using two-way (A) or one-way (B) ANOVA with Tukey’s multiple comparison test. Data are expressed as the mean ± SEM. ^#^ *p* < 0.05, ^##^ *p* < 0.01, ^###^ *p* < 0.001 compared with Nor; * *p* < 0.05, ** *p* < 0.01, *** *p* < 0.001 compared with HFHC. Nor, normal control group; HFHC, high-fat and high-cholesterol diet group; Sim, HFHC diet plus Sim group; SBH, HFHC diet plus SBH group.

To evaluate glucose tolerance, an oral glucose tolerance test (OGTT) was performed 10 weeks after the HFHCD, and the levels of blood glucose and their area under the curve (AUC) were measured at fasting and after the injection of glucose. The AUC value was calculated to quantify the OGTT. The AUC value depends on the rate of elimination of the administered glucose from the body. As shown in Figure 4A, in the HFHCD mice, there was a peak at 20 min, although at a higher level than in the Nor group. Compared with the HFHCD mice, Sim and SBH significantly lowered the OGTT level at 20 and 40 min. Similarly, the glucose AUC was significantly decreased due to Sim (*p* < 0.05) and SBH (*p* < 0.01) compared with the HFHCD-induced mice. These results demonstrated that the HFHCD mice had an impaired ability for glucose clearance, indicating glucose intolerance, and Sim or SBH administration improved HFHCD-induced glucose intolerance.

Plasma glucose-dependent insulinotropic polypeptide (GIP), glucagon-like peptide 1 (GLP-1), and insulin levels were analyzed using a multiplex immunoassay method (Figure 4B). GIP and GLP-1 promote glucose-dependent insulin secretion [20]. The plasma levels of GIP, GLP-1, and insulin were significantly increased in the HFHCD-induced mice, which was significantly reversed by Sim or SBH administration. SBH was expected to increase insulin secretion as glucose AUC decreases, but as a result, it was confirmed that it decreased insulin blood concentration due to inhibition of insulin receptor β-subunit protein expression. These results indicated that Sim and SBH administration lowered insulin secretion compared with the HFHCD-induced mice.

### 3.5. SBH Reversed HFHCD-Induced Regulation of Signaling Molecules

**Figure 5:**
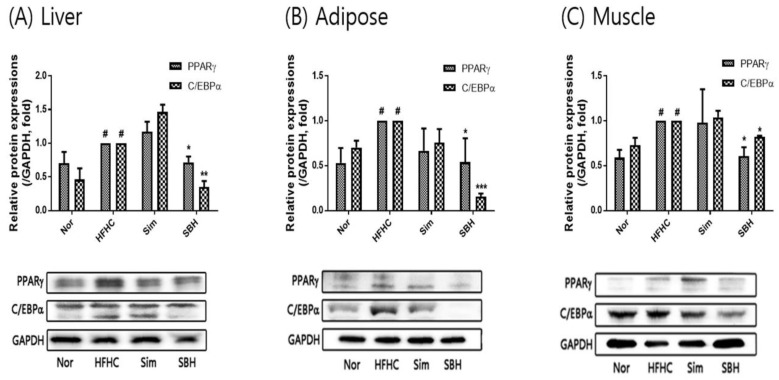
Expression of PPARγ and C/EBPα proteins in liver (**A**), adipose (**B**), and muscle (**C**) tissue. The number of experiments: Nor (*n* = 3), HFHC (*n* = 3), Sim (*n* = 3), SBH (*n* = 3). Data are expressed as the mean ± SEM. *^#^ p* < 0.05 compared with Nor; * *p* < 0.05, ** *p* < 0.01, *** *p* < 0.001 compared with HFHC. Nor, normal control group; HFHC, high-fat and high-cholesterol diet group; Sim, HFHC diet plus Sim group; SBH, HFHC diet plus SBH group.

Western blotting was performed to investigate the effect of SBH on the protein expression and/or phosphorylation of the adipogenic transcription factors PPARγ and C/EBPα in the liver, adipose tissue, and muscle of the HFHCD mice. As shown in Figure 5, PPARγ and C/EBPα expression was increased in the liver, adipose tissue, and muscle from HFHCD-induced mice compared with the Nor group. Herein, SBH administration showed a significant decrease in PPARγ and C/EBPα expression. However, there was no significant difference due to Sim. These results suggested that SBH administration prohibited adipogenesis by regulating PPARγ and C/EBPα.

### 3.6. SBH Reversed HFHCD-Induced Inhibition of AMPK and HSL Phosphorylation

**Figure 6:**
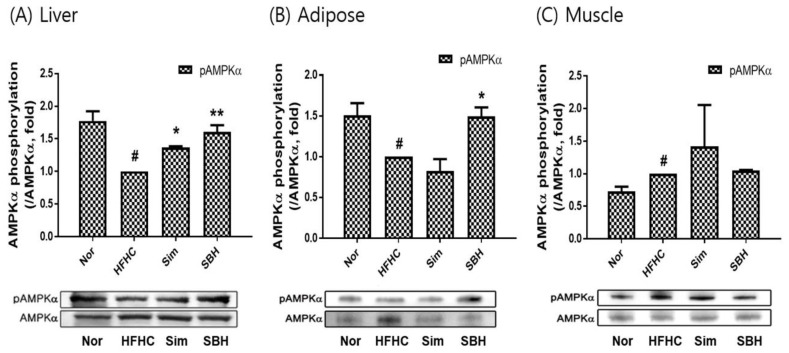
AMPK activity measured as phosphorylation of AMPKα in liver (**A**), adipose (**B**), and muscle (**C**) tissues. The number of experiments: Nor (*n* = 3), HFHC (*n* = 3), Sim (*n* = 3), SBH (*n* = 3). Data are expressed as the mean ± SEM. *^#^ p* < 0.05 compared with Nor; * *p* < 0.05, ** *p* < 0.01 compared with HFHC. Nor, normal control group; HFHC, high-fat and high-cholesterol diet group; Sim, HFHC diet plus Sim group; SBH, HFHC diet plus SBH group.

**Figure 7:**
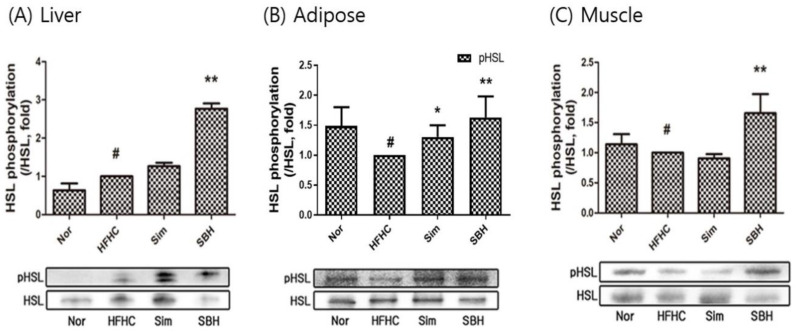
Expression of HSL phosphorylation in liver (**A**), adipose (**B**), and muscle (**C**) tissues. The number of experiments: Nor (*n* = 3), HFHC (*n* = 3), Sim (*n* = 3), SBH (*n* = 3). Data are expressed as the mean ± SEM. *^#^ p* < 0.05 compared with Nor; * *p* < 0.05, ** *p* < 0.01 compared with HFHC. Nor, normal control group; HFHC, high-fat and high-cholesterol diet group; Sim, HFHC diet plus Sim group; SBH, HFHC diet plus SBH group.

AMPKα is a key player in energy homeostasis, and its phosphorylation inhibits lipogenesis, leading to improvements in obesity-related traits [21]. In this study, AMPKα phosphorylation was assessed in the liver, adipose tissue, and muscle (Figure 6A–C). The HFHCD-induced mice inhibited AMPKα phosphorylation compared with the Nor group, while SBH administration significantly increased the phosphorylation, especially in the liver (*p* < 0.01) and adipose tissue (*p* < 0.05). Sim administration significantly increased the phosphorylation of AMPKα in the liver, but the adipose and muscle tissues showed no significant difference. In addition, we confirmed the phosphorylation expression of HSL, which is a lipolysis-related factor (Figure 7A–C). The protein level of phosphorylation of HSL significantly decreased in adipose and muscle tissue from HFHCD-induced mice, but not in the liver tissue. SBH administration significantly increased the phosphorylation of HSL levels in the liver, adipose tissue, and muscle from HFHCD-induced mice. Sim administration significantly increased the phosphorylation of HSL in the adipose tissue, but the liver and muscle tissue showed no significant difference. Therefore, we suggest that compared with Sim, SBH is likely to be more efficient in contributing to lipolysis.

### 3.7. SBH Reduced HFHCD-Induced Expression of Inflammatory Cytokines

**Figure 8:**
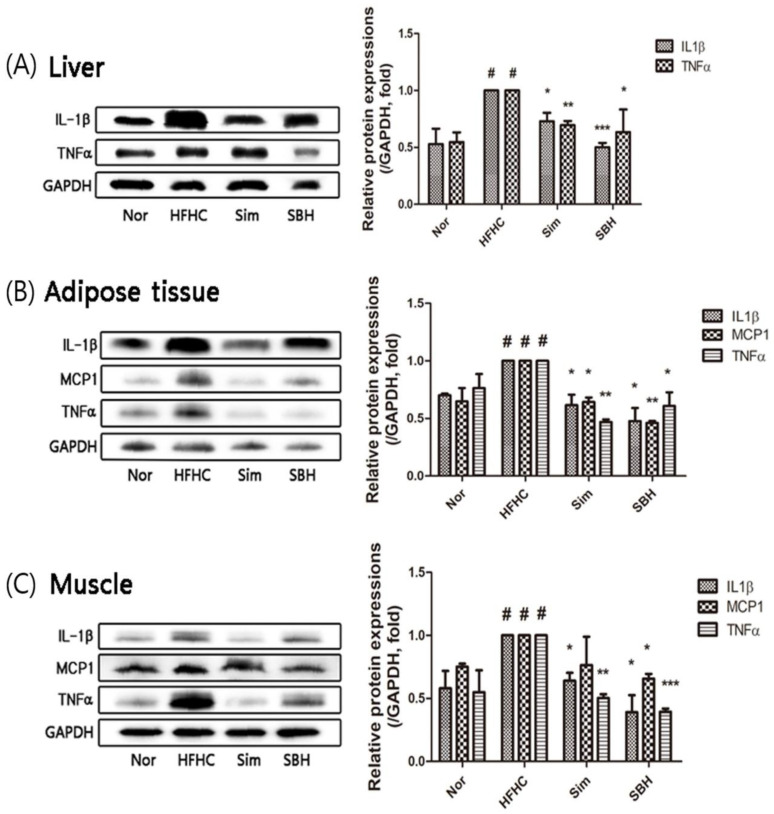
Effects of SBH on the expression of inflammatory cytokines. Western blot analyses of IL-1β and TNFα in the liver (**A**) and IL-1β, TNFα, and MCP1 in the adipose (**B**) and muscle (**C**) tissues were performed. Densitometric quantification was expressed as a fold change in the ratio of protein expression/GAPDH (an internal control). The number of experiments: Nor (*n* = 3), HFHC (*n* = 3), Sim (*n* = 3), SBH (*n* = 3). Data are expressed as the mean ± SEM. *^#^ p* < 0.05 compared with Nor; * *p* < 0.05, ** *p* < 0.01, *** *p* < 0.001 compared with HFHC. Nor, normal control group; HFHC, high-fat and high-cholesterol diet group; Sim, HFHC diet plus Sim group; SBH, HFHC diet plus SBH group.

AMPK has a key anti-inflammatory role in adipocytes by inhibiting HSL [6]. To investigate whether SBH can regulate inflammatory cytokine expression, levels of IL-1β, MCP1 (monocyte chemoattractant protein-1), and TNFα in the liver, adipose tissue, and muscle were compared between the four groups. The protein expression levels of these cytokines in HFHCD-induced mice were approximately two times higher than those in the Nor group (Figure 8A–C). The administration of SBH or Sim significantly decreased the expression levels of IL-1β, MCP1, and TNFα proteins in the liver, adipose tissue, and muscle compared with the HFHCD-induced mice, the levels of which appeared to be restored to that of the Nor group.

## 4. Discussion

According to the World Health Organization (WHO), obesity is defined as lipid accumulation and body weight gain [22], but it is also frequently accompanied by lipid accumulation in non-adipose tissues, such as the liver [23]. In our present study, the HFHCD-induced mice had increased body weight (Figure 2B) and liver and adipose tissue weights compared with Nor mice (Table 2). We also found steatosis in the liver (Figure 3A) and markedly elevated plasma levels of GOT, GPT, TC, TG, and LDL cholesterol (Figure 3B). In addition, increased impaired glucose tolerance significantly increased the plasma levels of GIP, GLP-1, and insulin. This was consistent with studies suggesting that dyslipidemia through obesity is related to weight distribution, insulin sensitivity, and impaired glucose tolerance [24]. Therefore, we confirmed that the HFHCD-induced mice exhibited a typical obesity phenotype, and the systemic effects of obesity were investigated by administering Sim and SBH. The Sim administration significantly reduced the HFHCD-induced mice body weight gain (Figure 2B) and showed inhibition of liver lipid accumulation, but no significant difference in plasma lipid contents. However, the administration of SBH significantly reversed the HFHCD-induced mice increases in plasma levels of TC, TG, and LDL cholesterol, as well as liver lipid accumulation (Figure 3). Changes in cretin hormones, including GIP and GLP-1, play an important role in glucose homeostasis as a cause of insulin secretion disorders and glucose metabolism abnormalities. In addition, as shown in Figure 4, both SBH and Sim significantly reversed the HFHCD-induced mice’s impaired glucose tolerance, plasma insulin, and increased levels of GIP and GLP-1. It is thought that SBH administration had the effect of improving obesity, insulin secretion, and dyslipidemia caused by an HFHCD, which was more effective than Sim administration.

Adipogenesis, which is the mature adipocyte-generating complex process, is highly regulated by transcriptional cascades and signaling pathways, including PPARγ and C/EBPα [25]. We showed that the protein expression of both transcription factors of PPARγ and C/EBPα in the liver, adipose, and muscle tissues of the HFHCD-induced mice was significantly increased, which was reversed by SBH treatment, whereas the Sim treatment showed no significant effect (Figure 5). AMPK, which is an AMP-dependent protein kinase, plays an important role in sensing energy homeostasis. The activation of AMPK emerged as a therapeutic target for obesity and is essential for the suppression of adipogenesis [26]. AMPK activation induces many metabolic changes, such as increased glucose uptake and metabolism by muscle and other tissues, decreased gluconeogenesis in the liver, decreased fatty acid synthesis, and increased oxidation [27]. Therefore, in this study, the HFHCD-induced mice significantly reduced their phosphorylation of AMPKα in the liver and adipose tissues, and it was observed that SBH administration increased the phosphorylation of AMPKα. However, Sim administration only had an effect on the liver tissue; there was no difference in the other tissues (Figure 6). From our results, it can be interpreted that SBH promoted fatty acid oxidation in the liver and adipose tissue. HSL, which is a multifunctional enzyme, is a major regulator of lipolysis and is expressed in a variety of tissues, including adipose tissue, muscle, and gonads [28]. In this study, the HFHCD-induced mice demonstrated a significant decrease in HSL phosphorylation, particularly in the adipocytes and muscle, but not in the liver tissue (Figure 7). However, the administration of SBH significantly increased HSL phosphorylation in the liver, adipose, and muscle tissue compared with the HFHCD-induced mice. On the other hand, Sim significantly increased this only in the adipose tissue. These results suggested that SBH stimulates lipolysis in the liver and adipose tissue by increasing the phosphorylation of HSL and AMPKα.

Excessive lipid accumulation was reported to induce insulin resistance in adipocytes, hepatocytes, and skeletal muscle cells by increasing the levels of various proinflammatory cytokines, including IL-1β, IL-6, TNFα, and MCP1 [29]. In this study, the HFHCD significantly increased the levels of the inflammatory factors IL-1β, TNFα, and MCP1 in the liver, adipose tissue, and muscle, which were significantly reduced by the Sim or SBH administration (Figure 8). Therefore, it was found that SBH has the potential to reduce inflammation induced by obesity.

In summary, aqueous extracts of SBH reduce plasma lipid content and have effects throughout the liver, adipose, and muscle tissue: they inhibit adipogenesis-related factors, promote lipolysis, and inhibit the secretion of inflammatory cytokines. This shows that the effect is similar to or more potent than the lipid-lowering drug simvastatin. Therefore, it is expected that SBH can be used as a treatment for obesity and related complications, but it is necessary to find out the exact mechanism of the physiologically active compound of SBH and the mechanism of anti-obesity.

## 5. Conclusions

In conclusion, SBH significantly lowered plasma TC, TG, and LDL-c levels; reduced liver fat accumulation; and decreased insulin secretion of the HFHCD-induced obese mice. In addition, SBH inhibited lipid accumulation by regulating the transcriptional factors and their downstream lipogenic targets via the activation of the AMPKα pathway in the liver, adipose, and muscle tissue. SBH also inhibited the secretion of inflammatory cytokines, such as IL-1β, TNFα, and MPC1.

These results indicated that SBH can be developed as a novel herbal medicine for the treatment of obesity and related complications without toxic effects.

## Figures and Tables

**Table 1:** Primary and secondary antibodies used for Western blotting.

Primary Antibody	Dilution	Catalogue No.	Company	Secondary Antibody	Dilution	Catalogue No.	Company
AMPKα	1:1000	sc-5298	Santa	Goat-anti-rabbit	1:5000	ADI-SAB-300	Enzo
C/EBPα	1:1000	sc-365318	Santa	Goat-anti-mouse	1:5000	ADI-SAB-100	Enzo
HSL	1:500	ab45422	Abcam	Goat-anti-rabbit	1:5000	ADI-SAB-300	Enzo
IL-1β	1:1000	12242s	Cell Signaling	Goat-anti-mouse	1:5000	ADI-SAB-100	Enzo
MCP1	1:1000	ab25124	Abcam	Goat-anti-rabbit	1:5000	ADI-SAB-300	Enzo
PPARγ	1:500	2430s	Cell Signaling	Goat-anti-rabbit	1:5000	ADI-SAB-300	Enzo
pAMPKα	1:500	2535s	Cell Signaling	Goat-anti-rabbit	1:5000	ADI-SAB-300	Enzo
pHSL	1:500	4139s	Cell Signaling	Goat-anti-rabbit	1:5000	ADI-SAB-300	Enzo
TNFα	1:1000	sc-52746	Santa	Goat-anti-mouse	1:5000	ADI-SAB-100	Enzo
GAPDH	1:1000	Sc-32233	Santa	Goat-anti-mouse	1:5000	ADI-SAB-100	Enzo

AMPKα, adenosine 5-monophosphate-activated protein kinase alpha; C/EBPα, cytidine-cytidine-adenosine-adenosine-thymidine (CCAAT) enhancer-binding protein alpha; HSL, hormone-sensitive lipase; IL-1β, interleukin-1 beta; MCP1, monocyte chemoattractant protein 1; PPARγ, peroxisome proliferator-activated receptor γ; pAMPKα, phospho-AMPKα; pHSL, phospho-HSL; TNFα, tumor necrosis factor alpha; GAPDH, glyceraldehyde-3-phosphate dehydrogenase; Santa Cruz Biotechnology, CA, USA; Cell Signaling Technology Inc., Danvers, MA, USA; Abcam, Cambridge, UK; Enzo Life Science, Farmingdale, NY, USA.

**Table 2:** Effect of SBH on the tissue weights in the HFHCD-induced mice.

Tissue Type	Nor	HFHC	Sim	SBH
**Liver**	1.288 ± 0.060	2.621 ± 0.284 ^###^	1.633 ± 0.079 ***	1.945 ± 0.083 *
**Adipose tissue**	0.618 ± 0.037	2.601 ± 0.158 ^###^	1.483 ± 0.061 **	1.694 ± 0.115 ^0.057^
**Kidney**	0.158 ± 0.026	0.181 ± 0.010	0.167 ± 0.021	0.182 ± 0.013

The tissue weights of the liver, adipose tissue, and kidney were measured. Significant differences between the groups were assessed using one-way ANOVA with Tukey’s multiple comparison test. The number of experiments: Nor (*n* = 8), HFHC (*n* = 7), Sim (*n* = 7), SBH (*n* = 7). ^##*#*^ *p* < 0.001 compared with Nor; * p < 0.05, ** p < 0.01, *** p < 0.001 compared with HFHC. Nor, normal control group; HFHC, high-fat and high-cholesterol diet group; Sim, HFHC diet plus Sim group; SBH, HFHC diet plusSBH group.

## Data Availability

Not applicable.
